# Artificial thymic organoid culture generates functional iPSC-derived CD4+ invariant natural killer T cells

**DOI:** 10.1038/s42003-025-09462-1

**Published:** 2026-01-09

**Authors:** Sara Shiina, Tatsuki Ueda, Shoichi Iriguchi, Yasushi Uemura, Shin Kaneko

**Affiliations:** 1 Department of Cell Growth and Differentiation, Shin Kaneko Laboratory, Center for iPS Cell Research and Application (CiRA), Kyoto University, Kyoto, Japan; 2 Takeda-CiRA Joint Program (T-CiRA), Fujisawa, Kanagawa Japan; 3 Division of Cancer Immunotherapy, Exploratory Oncology Research & Clinical Trial Center, National Cancer Center, Kashiwa, Japan; 4 Laboratory of Cancer Immunotherapy and Immunology, Transborder Medical Research Center, University of Tsukuba, Tsukuba, Japan

**Keywords:** Induced pluripotent stem cells, NKT cells, Cancer microenvironment, Regenerative medicine

## Abstract

Invariant NKT (iNKT) cells have a T-cell receptor that is common to all individuals and are activated by recognizing glycolipids on MHC class I-like CD1d molecules. Activated iNKT cells are known to exert anti-tumor effects through the activation of other immune cells and have attracted attention as promising T cells for eliciting anti-tumor immunity. However, securing a sufficient number of iNKT cells is an obstacle to treatment because iNKT cells are a very small cell population, less than 0.1% of the peripheral blood lymphocytes. Although previous studies have demonstrated redifferentiation of a large number of CD4^–^CD8^–^ double-negative iNKT cells from induced pluripotent stem (iPS) cells in two-dimensional monolayer cultures, CD4^+^ single-positive (CD4SP) iNKT cells could not be induced. Here we show CD4SP iNKT cells can be obtained by three-dimensional (3D) organoid culture (3D-CD4+ iNKT cell). We additionally describe 3D-CD4+ iNKT cells show antigen-specific helper functions, as they proliferate, produce interferon-γ/interleukin-4 (IFN-γ/IL-4), and induce dendritic cell maturation in response to α-galactosylceramide. Furthermore, they reverse the inhibition of T cell proliferation induced by immunosuppressive macrophages in an antigen-specific manner. Collectively, 3D-CD4+ iNKT cells may become an adjuvant T-cell source to enhance current T-cell immunotherapy against solid tumors.

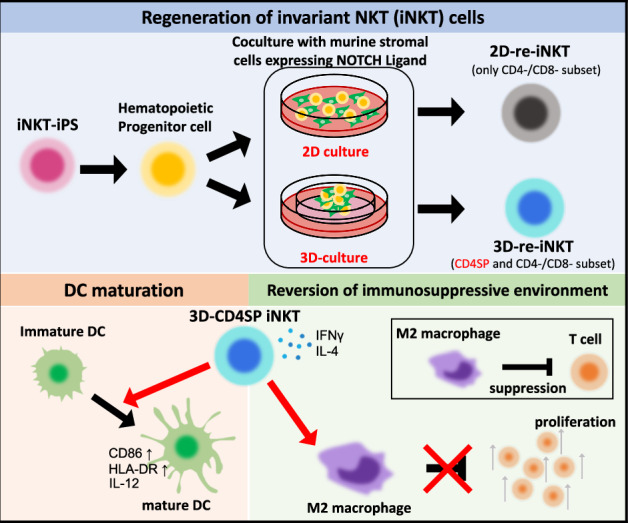

## Abstract (web summary)

Functional CD4+ invariant Natural Killer T cells were induced from iPSCs using the Artificial Thymic Organoid method. It showed antigen specific adjuvant function and ability to release T cell suppression by M2 macrophage.

## Introduction

Recently, immune checkpoint inhibitors and chimeric antigen receptor (CAR)-T cells have demonstrated therapeutic efficacy against some cancers, and are being developed for use in a wide variety of diseases. In particular, T-cell immunotherapy has shown significant efficacy against hematologic malignancies but not against solid tumors. One reason for this is the establishment of an immunosuppressive state within tumors owing to the infiltration and proliferation of immunosuppressive cells^1^. Efforts have been made to overcome the immunosuppressive environment by combining multiple modalities.

Therefore, iNKT cells are potential candidates for this purpose. iNKT cells are unique immune cells that exhibit both innate and acquired properties. In contrast to T cells with diverse TCR sequences, iNKT cells have invariant TCRα chains (human: Vα24-Jα18) and are activated by recognizing glycolipids presented on the MHC class I-like molecule CD1d^2^. There are two subsets of iNKT cells: CD4 single-positive iNKT cells (CD4^+^ iNKT) and CD4^−^CD8^−^ double-negative iNKT (DN iNKT) cells. Activated CD4^+^ iNKT cells induce dendritic cell (DC) maturation and production of IL-12p70 in DCs via production of Th1 and Th2 cytokines (e.g., IFN-γ and IL-4) and increased expression of CD40 ligand (CD40L)^3^. CD4^+^ iNKT cell activation ultimately leads to the activation of immune cells such as natural killer (NK) cells^4^, cytotoxic T-lymphocytes (CTLs)^5^, and helper T (Th) cells through their adjuvant functions. In contrast, DN iNKT cells eliminate DCs by cytolysis, markedly reduce IL-12p70 levels, and induce Th2 differentiation of naive Th cells^3^.

The anti-tumor potential of iNKT cells has been demonstrated in several clinical trials^6,7^. Infiltration of iNKT cells into the tumor tissue is associated with improved prognosis and survival^8,9^. These results indicate that the adoptive transfer of iNKT cells may improve anti-tumor efficacy of T-cell immunotherapy. However, iNKT cells comprise less than 0.1% of peripheral blood lymphocytes, and further reductions in cell number and function have been reported in cancer patients^10,11^. Furthermore, excessive proliferation of iNKT cells in vitro leads to a functional decline in iNKT cells. Therefore, obtaining iNKT cells from patients in sufficient numbers and with sufficient functions to induce effective anti-tumor immune responses is an obstacle for iNKT cell-based immunotherapy. Thus, studies have been conducted to produce iNKT cells of sufficient quality and quantity for therapy using iNKT cell-derived iPS cells.

We and other groups have previously reported the regeneration of iPS cells derived from human iNKT cells (iNKT-iPS cells)^12,13^. We chose to use iPS cells derived from iNKT cells because iNKT-iPS cells may present better potential to redifferentiate into iNKT cells due to epigenetic memory and rearranged T-cell receptor of the original iNKT cells as described in the previous studies using different somatic cell sources^14–16^. The iNKT-iPS cells were cultured on C3H10T1/2 cells to induce hematopoietic progenitor cells, which were then cultured on an OP9-DL1 monolayer to induce iNKT cell differentiation (2D-re-iNKT cells). This differentiation method yielded CD4^−^/CD8^−^ (DN) cells and CD8 single-positive cells. However, other major subsets of iNKT cells, such as CD4^+^ iNKT cells, cannot be obtained using this method. In addition, the 2D-re-iNKT cells did not express CD5, a marker of primary mature iNKT cells. Furthermore, they exhibited several properties that differed from those of primary iNKT cells, such as a relatively lower proliferative capacity and limited cytokine-producing ability. As mentioned above, CD4^+^ iNKT cells actively participate in anti-tumor activity, and regeneration of CD4^+^ iNKT cells from iPS cells is a prerequisite for successful iNKT cell-based immunotherapy.

We hypothesized that the adaptation of three-dimensional organoid cultures during T-cell differentiation would lead to the generation of functional CD4^+^ iNKT cells as well as CD5^+^ DN iNKT cells from iNKT-iPS cells, because it was recently reported that CD4-positive αβTCR-positive T cells can be generated by making a cell aggregate comprising DL1-expressing mouse feeder cells and iPS cell-derived mesoendodermal progenitor cells and placing them in an air-liquid interface culture environment (Artificial Thymic Organoid: ATO)^17,18^. Here, we show that ATO-mediated redifferentiation of iNKT-iPS cells can regenerate both CD4^+^ and DN/CD5^+^ iNKT cells with much better functionality than 2D-re-iNKT cells. This report demonstrating Vα24-Jα18 TCR dependent and antigen-specific helper functions of CD4^+^ iNKT cells derived from iPS cells.

## Result

### Induction of iNKT-cell differentiation from iNKT-iPS cells using ATO method

**Fig. 1:**
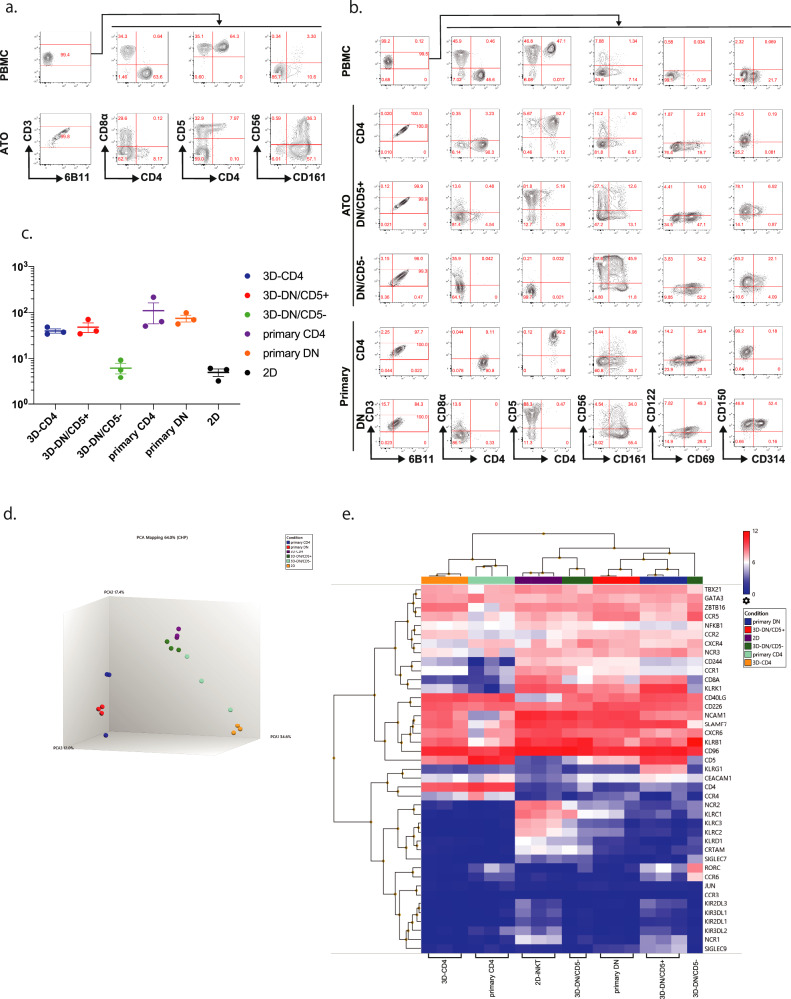
Induction of iNKT-cell differentiation from iNKT-iPS cells using ATO method. **a** Representative flow cytometry plots of re-differentiating T cells from CD4^+^ iNKT-iPSCs using the ATO method (n = 5) (gated by CD45, CD3 and 6B11). PBMC stands for peripheral blood mononuclear cells. **b** Representative flow cytometry plots of each fraction of 3D-re-iNKT and primary iNKT cells after TCR stimulation (gated by CD45, CD3 and 6B11). (3D-re-iNKT: n = 5; primary iNKT: n = 3) **c** Fold expansion of primary CD4^+^/DN iNKT cells, 2D-re-iNKT cells and newly induced 3D-re-iNKT cells around 2 weeks after stimulation with α-GalCer-pulsed PBMCs. Results obtained from three independent experiments. Data represent mean ± SEM of n independent experiments. Significance was assessed by one-way ANOVA with Tukey’s multiple comparison test. **d** Principal component analysis (PCA) of the transcriptomic data of each subset of primary iNKT cells, 2D-re-iNKT cells and 3D-re-iNKT cells. (primary iNKT: n = 3; 2D-re-iNKT: n = 3; 3D-re-iNKT: n = 3;) **e** Gene expression profiles of primary iNKT cells, 2D-re-iNKT cells and 3D-re-iNKT cell subsets (primary iNKT: n = 3; 2D-re-iNKT: n = 3; 3D-re-iNKT: n = 3;). Hierarchical clustering was performed based on gene profiles related to transcription factors, NK activating and inhibitory receptors, and tissue inflammatory homing markers.

We induced iNKT cell differentiation from iNKT-iPS cell-derived hematopoietic progenitor cells using ATO (3D-re-iNKT cells). The iNKT cell-derived iPS cells were previously established from peripheral blood CD4^+^ iNKT cells^12^. The iPS cells were cultured in a feeder-free culture system for 14 days to generate hematopoietic progenitor cells as previously reported^19^. The hematopoietic progenitor cells obtained were cultured with MS5DL4 cells in the form of a cell pellet on a cell culture insert. Flowcytometric analysis after 5 weeks showed that they uniformly expressed invariant TCR (stained with monoclonal antibody (mAb) 6B11 specific for invariant Vα24-Jα18 CDR3) and CD161, an NK receptor. CD4SP cells were also present, most of which were CD5 positive (Fig. 1a).

In addition to the CD4SP and DN/CD5^+^ fractions (3D-CD4^+^ iNKT and 3D-DN/CD5^+^ iNKT cells), which are major subsets of peripheral blood iNKT cells^2^, 3D-re-iNKT cells contained a DN/CD5^−^ fraction (3D-DN/CD5^−^ iNKT cells) with an expression similar to that of 2D-re-iNKT cells. To compare the functions of each fraction, each of these three fractions was separated, stimulated with the synthetic sphingoglycolipid αGC^20^, and analyzed for surface antigens. Most 3D-CD4^+^ and -DN/CD5^+^ iNKT cells expanded, while maintaining CD5 expression. In addition, 3D-re-iNKT cells expressed CD150 (SLAM) (Fig. 1b). These findings demonstrate successful regeneration and expansion of CD4^+^, DN/CD5^+^, DN/CD5^−^ fractions from iNKT-iPS cells by ATO method. The CD8SP fraction was also obtained, but stimulation with aGC resulted in almost no cell growth and no CD8 expression (Supplementary Fig. 1a-c). Therefore, further characteristics analysis of 3D-CD8-iNKT cells could not be performed.

Next, we evaluated the proliferative ability of each subset of 3D-re-iNKT and primary iNKT cells in response to αGC stimulation (Fig. 1c). 3D-CD4^+^ and -DN/CD5^+^ iNKT cells showed proliferation rate comparable to primary iNKT cells. On the other hand, 3D-DN/CD5-iNKT cells and 2D-re-iNKT cells tended to show lower proliferation rates compared to the other two fractions and primary iNKT cells, although there was no significant difference. These results are consistent with our previous report that 2D-re-iNKT cells, showed low proliferation rates (7.5–24.1 fold) in response to αGC stimulation.

We further analyzed the global gene expression profiles of primary iNKT cells, 2D-re-iNKT cells and each subset of 3D-re-iNKT cells. Principal component analysis (PCA) of the transcriptomic data indicated although 3D-CD4 and primary CD4+ iNKT were allocated in different position, they were much closer than 2D-re-iNKT cells (Fig. 1d). Hierarchical clustering of the transcriptomes enabled by mRNA sequencing of the selected genes showed that 3D-DN/CD5^−^ iNKT cells are close to 2D-re-iNKT cells with the same phenotype, and the ATO-specific subsets 3D-CD4^+^ cells and primary CD4^+^ iNKT cells are in the same cluster (Fig. 1e). 2D-re-iNKT and 3D-DN/CD5^−^ iNKT cells expressed high levels of NK-activating receptor genes (e.g., *NCAM1*, *NCR1*, *NCR2*, *KLR2*, and *KLR3*), indicating that they were NK cell-like cells. 2D-re-iNKT cells also expressed high levels of NK inhibitory receptor genes (e.g., *KIR3DL1*, *KIR3DL2*, *KIR2DL1*, and *KIR2DL2*), suggesting that they are particularly similar to NK-like cells. Interestingly, 3D-CD4^+^ iNKT cells expressed high levels of CCR4, which is primarily expressed in IL-4-producing CD4^+^ iNKT cells^21–23^.

### Cytotoxic activities of 3D-re-iNKT cells

**Fig. 2:**
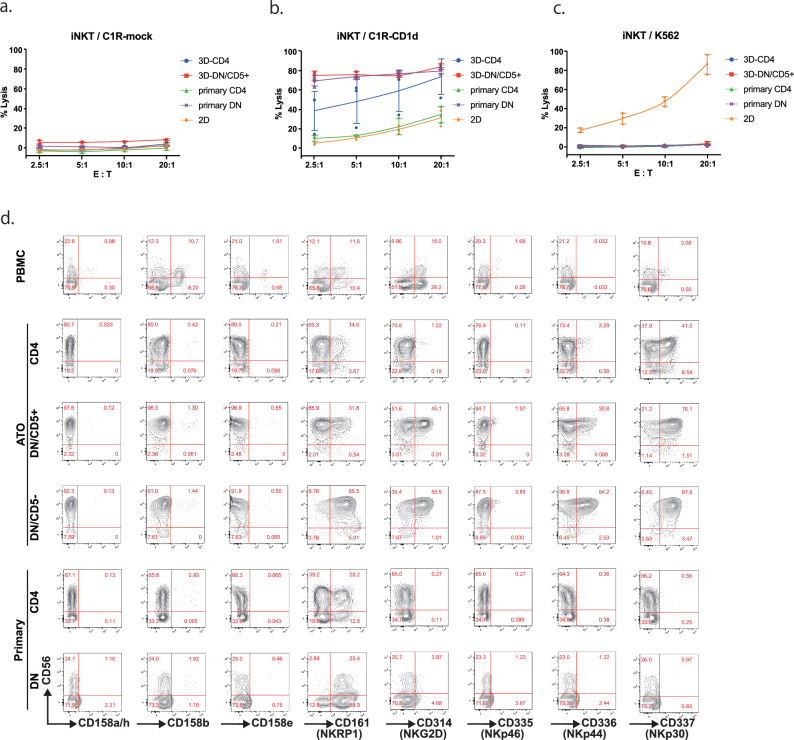
Cytotoxic activities of 3D-re-iNKT cells. **a** In vitro cytotoxicity assay of primary iNKT cells, 2D-re-iNKT cells and 3D-re-iNKT cells measured by non-radioactive cytotoxicity assay using C1R-CD1d cells as target cells. Representative data of three (3D-re-iNKT and primary iNKT cells) or two (2D-re-iNKT cells) independent experiments are shown. Dots represent individual values. Means are connected by lines; error bars represent SD. n  =  3 per point. **b** In vitro cytotoxicity assay of primary iNKT cells, 2D-re-iNKT cells and 3D-re-iNKT cells measured by non-radioactive cytotoxicity assay using C1R-mock cells as target cells. Representative data of three independent experiments are shown. Dots represent individual values. Means are connected by lines; error bars represent SD. n  =  3 per point. **c** In vitro cytotoxicity assay of primary iNKT cells, 2D-re-iNKT cells and 3D-re-iNKT cells measured by non-radioactive cytotoxicity assay using K562 cells as target cells. Representative data of three independent experiments are shown. Dots represent individual values. Means are connected by lines; error bars represent SD. n  =  3 per point. **d** Expression of NK cell receptors in 3D-re-iNKT cells and primary iNKT cells (gated by CD45, CD3, and 6B11).

Next, we tested the antigen-specific cytotoxicity of 3D-re-iNKT cells. Since iNKT cells are activated by recognizing glycolipids present on the MHC class I-like molecule CD1d, we co-cultured iNKT cells with C1R-CD1d and C1R-mock cells and evaluated their cytotoxicity in the presence of the glycolipid αGC. 2D-re-iNKT cells, 3D-re-iNKT cells and primary iNKT cells all exhibited weak baseline cytotoxic activity when cocultured with C1R-mock (Fig. 2a). When co-cultured with C1R-CD1d, 3D-CD4^+^ iNKT cells showed intermediate cytotoxicity, whereas primary CD4^+^ iNKT cells showed low cytotoxicity. Under the same conditions, 3D-DN/CD5^+^ iNKT cells showed strong cytotoxicity, similar to that of primary DN iNKT cells. On the other hand, 2D-iNKT cells showed low cytotoxicity despite being DN iNKT cells (Fig. 2b).

We also examined antigen-independent cytotoxicity of 3D-re-iNKT cells by co-culture them with K562, a cell line used to test antigen-independent cytotoxicity. Only 2D-re-iNKT cells showed strong cytotoxic activity against K562, whereas 3D-CD4+ and 3D-DN/CD5+ iNKT cells did not show cytotoxic activity, similar to primary iNKT cells (Fig. 2c)^24^. To identify the possible reasons for the different antigen-independent cytotoxicity, we compared the expression of NK-activating and inhibitory receptors in each subset (Fig. 2d). NKp44, a natural cytotoxicity receptor, and NKG2D were negative in primary iNKT cells and 3D-CD4^+^ iNKT cells, while 3D-DN/CD5^+^ and -DN/CD5^−^ iNKT cells expressed these receptors. Thus, the level of antigen-independent nonspecific cytotoxicity against K562 cells could be partly explained by the differential NK-activating receptors. These results were consistent with the global gene expression profiles.

### Helper functions of iNKT cells: maturation of dendritic cells

iNKT cells can indirectly promote the activation of CTLs and NK cells through DC activation. CD40L molecules expressed on activated iNKT cells stimulate DCs through CD40 ligation, inducing the upregulation of DC maturation markers and the production of IL-12p70^2^. IL-12p70 is a potent NK cell activator and an important inducer of functional antigen-specific Th1 cells and CTL responses factor^25^.

**Fig. 3:**
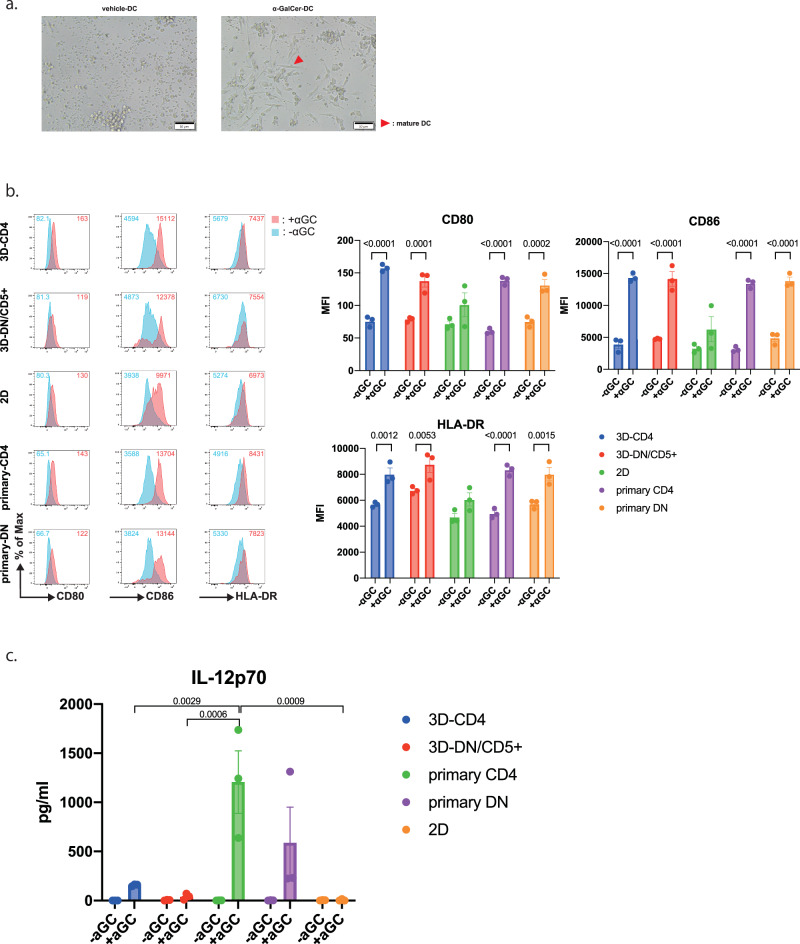
Helper functions of iNKT cells : maturation of dendritic cells. **a** Brightfield images after 24 h co-culture of vehicle-DCs or α-GalCer-DCs with 3D-CD4^+^ iNKT cells at an effector-to-target (E:T) ratio of 1:1. The cell pointed by red triangle indicate morphological example of mature DC. **b** The histograms represent the mean fluorescent intensity of DC maturation markers (CD80, CD86 and HLA-DR) obtained from vehicle-treated control in blue and aGC-treated group in red in the presence of iNKT cells. Bar graphs is summarizing the mean fluorescent intensities of DC maturation markers for the three trials. Data represent mean ± SEM of n independent experiments. Significance was assessed by 2way ANOVA with Šídák’s multiple comparison test. **c** IL-12p70 production by DCs stimulated with primary iNKT cells, 2D-re-iNKT cells and 3D-re-iNKT cells under conditions with or without aGC. Data represent mean ± standard error from three independent experiments. Significance was assessed by 2way ANOVA with Tukey’s multiple comparison test.

**Fig. 4:**
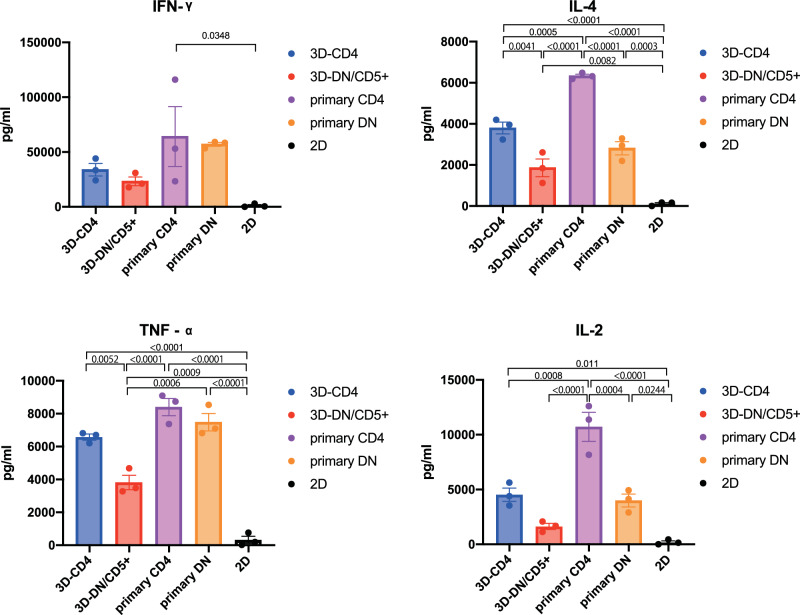
Helper functions of iNKT cells : cytokine profile of 3D-re-iNKT cells. Cytokine productions of primary iNKT cells, 2D-re-iNKT cells and 3D-re-iNKT cells co-cultured with DCs in the presence of vehicle or 100 ng/ml α-GalCer. Data represent mean ± standard error from three independent experiments. Significance was assessed by one-way ANOVA with Tukey’s multiple comparison test.

We first co-cultured each subset of 3D-re-iNKT cells with αGC pulsed-DCs and non-pulsed (vehicle)-DCs derived from peripheral blood mononuclear cells (PBMCs). After 24 h, microscopic observation revealed the presence of spindle-shaped DCs co-culture with αGC-DCs (Fig. 3a). In addition, flow cytometry analysis of DC maturation markers revealed that the expression of the costimulatory molecules CD80 (B7-1)/CD86 (B7-2) and HLA-DR increased in the presence of αGC in both 3D-re-iNKT cells and primary iNKT cells (Fig. 3b). We measured the concentration of IL-12p70 in the culture supernatants. In the co-culture with primary iNKT cells, IL-12p70 production was detected only when DCs were co-cultured with primary CD4^+^ iNKT cells. Similarly, in 3D-re-iNKT cells, IL-12p70 production was confirmed only when co-cultured with 3D-CD4^+^ iNKT cells. In contrast, co-culture with 2D-re-iNKT cells did not produce IL-12p70 (Fig. 3c). Next, we examined the cytokines produced by iNKT cells co-cultured with DCs. Each subset of 2D-re-iNKT cells, 3D-re-iNKT cells and primary iNKT cells was co-cultured with DCs and the cytokines in the supernatant were measured. Compared with 3D-DN/CD5^+^ iNKT cells, 3D-CD4^+^ iNKT cells produced higher amounts of cytokines, which was also true for primary iNKT cells. On the other hand, 2D-re-iNKT cells produced almost no cytokines compared with 3D-re- and primary iNKT cells (Fig. 4). Collectively, these results suggest that 3D-re-iNKT cells, especially 3D-CD4^+^ iNKT cells, have adjuvant functions closer to those of primary iNKT cells than 2D-re-iNKT cells.

### CD4^+^ iNKT cells can accelerate T-cell proliferation inhibited by alternatively activated macrophages in vitro

**Fig. 5:**
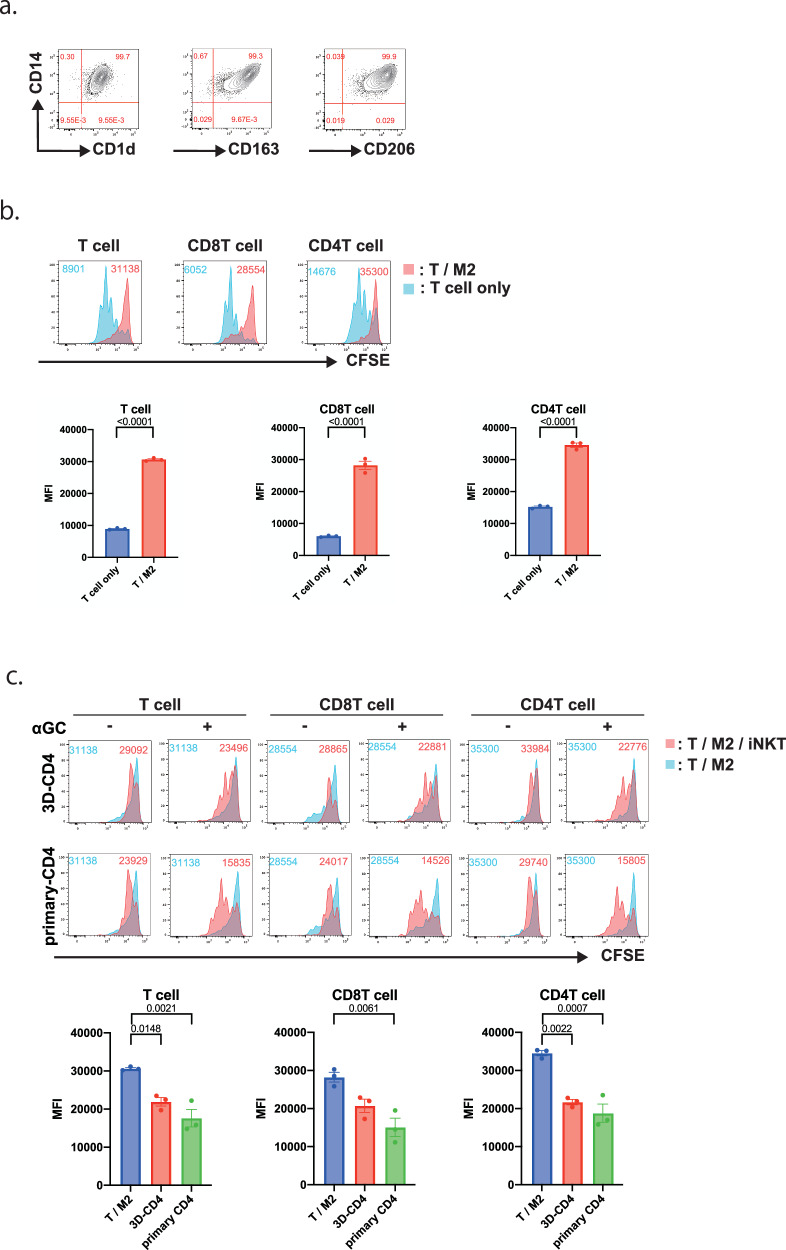
CD4 + iNKT cells can accelerate T-cell proliferation inhibited by alternatively activated macrophages in vitro. **a** Representative flow cytometry plots of PBMC-derived M2 macrophages showing expression levels of CD14 vs. CD1d, CD163, and CD206 (gated on live cells). **b** Representative histograms showing the cell division of CFSE-labeled T cells (left), CD8 T cells (middle), and CD4 T cells (right) co-cultured with (red) or without M2 macrophages (blue) for 3 days (gated by CD3 + , Vα24−, and CFSE + ). Division of T cells activated by CD3/CD28 beads is shown. Bar graphs is summarizing the mean fluorescent intensities of CFSE for the three trials. Data represent mean ± SEM of n independent experiments. Significance was assessed by unpaired t-test. **c** Representative histograms showing cell division of CFSE-labeled T cells (left two panels), CD8 T cells (middle two panels), and CD4 T cells (right two panels) co-cultured with M2 and primary CD4/3D-CD4^+^ iNKT cells (red) or with only M2 macrophages (blue) for 3 days (gated by CD3 + , Vα24−, and CFSE + ). Division of T cells activated by CD3/CD28 beads is shown. + and − at the top of each column indicate the presence or absence of αGC, respectively. Bar graphs is summarizing the mean fluorescent intensities of CFSE for the three trials. Data represent mean ± SEM of n independent experiments. Significance was assessed by one-way ANOVA with Tukey’s multiple comparison test.

Macrophage activation is broadly classified into immunopromoting (M1) and immunosuppressive (M2) types. Their activation status are plastic and strongly influenced by their exposure to the surrounding environment, especially cytokines such as IFNγ^26^. Tumor-associated macrophages (TAMs) are abundant in tumors and play an important role in establishing the immunosuppressive environment of tumors because they are M2-type immunosuppressive cells. TAMs express CD1d, which is involved in the recognition mechanism of iNKT cells, and is considered one of the target cells for the anti-tumor effects of iNKT cell therapy^27^. Therefore, we investigated whether 3D-re-iNKT cells could improve the immunosuppressive environment by acting on M2 macrophages. First, M2 macrophages were induced from PBMCs and analyzed for surface antigens using flow cytometry. The induced M2 macrophages expressed CD1d and M2 macrophage markers such as CD163 and CD206 (Fig. 5a)^26^. Next, T cells isolated from the same donor PBMCs as the M2 macrophages were stained with CFSE and co-cultured with M2 macrophages. We evaluated whether M2 macrophages act as immunosuppressive cells by examining their effects on the proliferative activity of T cells. We found that CD8 T cell proliferation was markedly suppressed in the presence of M2 macrophages compared to that in T cells alone (Fig. 5b). Next, the effects of 3D-CD4^+^ iNKT cells on M2 macrophages were examined by co-culturing T cells, M2 macrophages, and 3D-CD4^+^ iNKT cells. In both 3D-CD4^+^ and primary iNKT cell co-culture conditions, suppression of T cell proliferation by M2 macrophages was not observed, and T cell proliferation was promoted. Furthermore, this effect was observed only in the presence of αGC (Fig. 5c). This suggests that 3D-CD4+ cells, similar to primary iNKT cells, were activated via CD1d on M2 macrophages and function to promote T cell division that have been suppressed by M2 macrophages.

## Discussion

In this study, we induced the re-differentiation of iNKT cells using a 3D culture method and analyzed their functions. Previously, the redifferentiation of iNKT cells was induced by co-culturing them with OP9DLL1 cells in a two-dimensional culture, as has been performed for T cells. However, CD4^+^ iNKT cells that exhibit an adjuvant effect, which is an important function of iNKT cells, could not be obtained using this method. Cells obtained by 2D culture were also negative for CD5, which is constantly expressed in mature T cells. In this study, we applied the ATO method to induce the differentiation of iNKT cells and succeeded in obtaining functional 3D-CD4^+^ iNKT cells. Functional evaluation of 3D-CD4^+^ iNKT cells revealed that exhibited antigen-specific adjuvant effects and the ability to release the T cell growth-suppressive function of M2 macrophages.

Using the ATO method, T cell induction from umbilical cord blood and iPS cells have been reported^17,18^. CD5-positive CD8SP and CD4SP cells were obtained from both patients. In conventional two-dimensional culture differentiation, only DP cells can be obtained, and CD8SP cells can be obtained by stimulating DP cells with CD3 antibodies. Thus, the ATO method is considered to better recapitulate physiological T-cell differentiation than conventional 2D differentiation methods. Unlike T-cells, iNKT cells differentiate via a unique pathway^28^. When T-cell progenitors acquire Vα24 invariant TCR at the DP stage, they become sensitive to stimulation by specific glycolipid antigens presented by the nonpolymorphic molecule CD1d. In addition, they become sensitive to self-stimulation by CD150, also known as SLAM (Signaling Lymphocyte Activation Molecule)^29–31^. The expression of CD150 is higher in 3D-re-iNKT cells than in 2D-re-iNKT cells, and CD4SP- and CD5-positive cells were obtained, suggesting that the ATO method is capable of producing more physiologically similar differentiated cells than conventional 2D culture methods in the case of iNKT cell differentiation. The possible reasons for obtaining CD8SP/CD4SP cells using the ATO method include the effect of the 3D structure on cell-cell interactions and the strength of Notch signaling; however, the detailed molecular mechanisms have not yet been determined and are points for further study. It is also worth mentioning that ATO differentiation cultures favor generation of CD8SP over CD4SP, even though iPS cells were established from CD4^+^ iNKT cell clone. Elucidating the mechanisms governing this phenomenon require further investigation.

Clinical studies using αGalCer-pulsed DC therapy in cancer patients have shown that CD4^+^ iNKT subsets of primary iNKT cells increase in the body early after administration, followed by an increase in DN iNKT subsets over time^32^. It has been reported that the CD4^+^ iNKT subset elicit DC-mediated activation of CTLs and NK cells, while the DN iNKT subset suppress their activations^3^. Therefore, the selective administration of CD4^+^ iNKT cells may induce more effective anti-tumor immune responses. However, as has been pointed out for (CAR)-T cell therapy, it is expected to be more difficult to obtain a therapeutic cell population from patients with cancer for iNKT therapy. Indeed, in our study, we were able to sufficiently expand iNKT cells from only one donor among multiple healthy donors. Recently, studies have reported on HSC-iNKT cells induced from cord blood-derived HSCs transfected with iNKT TCR using the ATO method; however, HSC-iNKT cells are almost exclusively CD4-negative cells^33^. Therefore, a new therapeutic option is to generate large numbers of CD4-positive iNKT cells using iPS cells. Thus, 3D-CD4^+^ iNKT cells are promising candidates for developing this therapeutic option. Interestingly, 3D-CD4^+^ iNKT cells not only have a higher proliferation capacity than 2D-re-iNKT cells, but also can be expanded in a donor-independent manner compared to primary iNKT cells. Thus, 3D-CD4^+^ iNKT subsets are superior in that they can ensure stable and large numbers of CD4^+^ iNKT subsets that play an important role in anti-tumor immune responses.

This study showed that 3D culture not only induced CD4-positive iNKT cells, but also improved their quality. For example, the adjuvant effect was improved. 3D-CD4^+^ iNKT cells produced IL-4, which was not produced by 2D-re-iNKT cells, and more immunostimulating capacity can be expected due to the synergistic effect with IFN-γ. To compare DC maturation ability, 2D-re-iNKT cells were examined for DC maturation ability by co-culturing iNKT:DC at a 1:10 cell ratio. In the case of 3D-re-iNKT cells, DC maturation was possible even at a cell ratio of iNKT:DC = 1:1. This improvement in the adjuvant effect may also be due to a reduction in nonspecific cytotoxicity. Elucidating the mechanism by which 3D-CD4 iNKT cells facilitate DC maturation requires further investigation, but the RNA-seq analysis in this study suggested interactions between CD40 and CD40LG could be a mechanism. In addition, higher amount of IL-4 production capacity by the 3D-CD4 iNKT cells compared to those by 2D-iNKT cells may contribute to promote the DC maturation we observed in this study. Primary CD4^+^ iNKT cells showed no NK cell-like cytotoxicity, whereas 2D-re-iNKT cells showed high non-specific cytotoxicity. In contrast, 3D-CD4^+^ iNKT cells showed no NK cell-like cytotoxicity, similar to primary CD4^+^ iNKT cells, suggesting that they can efficiently mature DCs without killing them. In a previous study, 2D-re-iNKT cells showed high non-specific cytotoxicity and expressed NKG2D, NKp44, and NKp46. Among the 3D-re-iNKT cells, 3D-DN/CD5^−^ iNKT cells showed high NK cell-like cytotoxicity, NK receptor expression, characters of which were similar to those of 2D-re-iNKT cells. On the other hand, 3D-CD4^+^ iNKT, 3D-DN/CD5^+^ iNKT cells showed lower NK cell-like cytotoxicity and NK receptor expression than 3D-DN/CD5^−^ iNKT cells, suggesting that the ATO method can be used to obtain cells with properties more similar to primary iNKT cells.

In addition, we examined the effects of 3D-CD4^+^ iNKT cells on immunosuppressive cells. Although CAR T-cell therapy, which is currently the focus of much attention, has shown remarkable efficacy in hematologic malignancies, it is less effective in solid tumors. One reason for this is the immunosuppressive environment of tumors^34^. We demonstrated that 3D-re-iNKT cells promoted T cell proliferation by removal of the inhibitory effect of TAMs on T cells. However, the mechanism of TAM regulation by 3D-CD4^+^ iNKT cells remains unclear and requires further investigation. One possible mechanism is the transformation of TAMs to inflammatory M1 macrophages by cytokines produced by CD4^+^ iNKT cells, such as IFNγ. Whether the CD4+ iNKT cells we generated can polarize toward Th1 and Th2 programs via DCs warrants further investigation as described in a previous study^35^. Other mechanism may include direct killing of M2 macrophages by iNKT cells because the M2 macrophages we induced expressed CD1d. Since suppressor macrophages can be induced from iPS cells^36^, the use of iPS cells is expected to elucidate more detailed mechanisms for the deregulation of TAMs.

In this study, we report the development of 3D-CD4^+^ iNKT cells that exhibit helper functions inherent to iNKT cells. Using this 3D culture method, we successfully induced the differentiation of large numbers of iNKT cells with high proliferative capacity from iPS cells. Furthermore, 3D-CD4^+^ iNKT cells are expected to be applied in more effective solid tumor therapies because of their adjuvant function and ability to improve the tumor microenvironment.

## Methods

### Preparation of Vα24 iNKT Cells and iNKT-iPSCs

All researches using human samples were approved by the Kyoto University School of Medicine ethical committee (no. G590) and conducted in compliance with the Declaration of Helsinki. Consent has been obtained from the donors of the cells.

Vα24 iNKT cells: PBMCs from three healthy donors were stimulated with irradiated PBMCs pulsed with α-galactosylceramide (α-GalCer; KRN7000) (10 ng/mL, Funakoshi, Tokyo) in the presence of 5 ng/mL rhIL-7 and 5 ng/mL rhIL-15 (Peprotec, UK), after which Vα24^+^ cells were sorted using flow cytometer. After 24 h, 20 U/mL recombinant human IL-2 (rhIL-2) was added. After 14 d, Vα24^+^6B11^+^CD4^−^CD8β^−^CD5^−^ or Vα24^+^6B11^+^CD4^−^CD8β^−^CD5^+^ or Vα24^+^6B11^+^CD4^+^CD8β^−^CD5^+^ cells were sorted using flow cytometer and were re-stimulated with irradiated PBMCs pulsed with α-GalCer in the presence of rhIL-7 and rhIL-15. After 24 h, rhIL-2 was added.

iNKT-iPSCs: iNKT-iPSCs were generated from iNKT cells using Sendai virus reprogramming vectors, as previously described^12^. iPSCs were maintained in StemFit AK02N (Ajinomoto, Japan) on iMatrix-511 (Matrixome, Japan), as previously described^37^.

### Cell lines

C1R transfectants were established as previously described^3^ and maintained in RPMI1640 supplemented with 10% FBS. The myelogenous leukemia cell line K562 was purchased from JCRB (Lot.08212012) and maintained in RPMI1640 supplemented with 10% FBS. To generate MS5-hDLL4 cells, MS5 cells were transduced with a retroviral vector encoding full-length human DLL4 as previously described^38^. The highest 5% of DLL4-expressing cells was sorted by FACS using an anti-DLL4 antibody and passaged in DMEM/10% fetal calf serum (FCS). Stable expression was confirmed by flow cytometry for DLL4 expression after several weeks of culture.

### Generation of HPCs from iPSCs

To initiate differentiation, iPSCs were expanded for 6–7 d on iMatrix-511 in StemFit AK02N and dissociated into single cells using 0.5× TryPLE Select (Thermo Fisher Scientific). A total of 3–6 × 10^5^ cells were resuspended in StemFit AK02N supplemented with 10 μM Y-27632 (FujiFilm Wako) and 10 μM CHIR99021 (Tocirs Bioscience) and cultured in 6-well ultra-low attachment plates (Corning) for 24 h. After 24 h, the EBs were collected, settled down to the bottom of the tube, and resuspended in 2 mL StemPro-34 (Thermo Fisher Scientific) supplemented with 10 ng/mL penicillin/streptomycin (Sigma), 2 mM Glutamax (Thermo Fisher Scientific), 50 μg/mL ascorbic acid-2-phosphate (Sigma), 400 μM monothioglycerol (MTG, Nacalai), and 100× Insulin-Transferrin-Selenium solution (ITS-G, Thermo Fisher Scientific) (referred to as EB basal medium), 50 ng/mL recombinant human (rh) BMP-4 (R&D Systems), 50 ng/mL rhVEGF (R&D Systems), and 50 ng/mL bFGF (FujiFilm Wako) per well. After 24 h, 6 μM SB431542 (FujiFilm Wako) was added. After 4 d, differentiating EBs were collected, washed, resuspended in 2 mL EB basal medium supplemented with 50 ng/mL rhVEGF, 50 ng/mL rhbFGF, and 50 ng/mL rhSCF (R&D Systems) per well, and cultured for 2 d. After 7 d, differentiating EBs were collected, washed, and resuspended in 2 mL EB basal medium supplemented with 50 ng/mL rhVEGF, 50 ng/mL rhbFGF, 50 ng/mL rhSCF, 30 ng/mL rhTPO (PeproTech), and 10 ng/mL FLT3L (PeproTech) per well. From day 7, differentiating cultures were collected and replaced with fresh day 7 medium every 2–3 d. Cultures were maintained in a 5% CO_2_/5% O_2_/90% N_2_ environment for the first 7 d and in a 5% CO_2_ environment from day 7 onwards.

### Differentiation of iNKT cells

iNKT-HPCs were differentiated into iNKT cells in ATO culture for five weeks. ATO was generated following a previously established protocol^17^ with slight modification. MS5-DLL4 cells were harvested by trypsinization and resuspended in serum free ATO culture medium composed of RPMI 1640 (Corning), 4% B27 supplement (ThermoFisher Scientific), 50 μg/mL ascorbic acid-2-phosphate (Sigma-Aldrich), 1% glutamine/penicillin/streptomycin (Sigma-Aldrich), 1% Glutamax (ThermoFisher Scientific), 5 ng/mL rhFLT3L and 5 ng/mL rhIL-7 (Peprotech). ATO culture medium was made fresh weekly. Depending on the experiment, 1.5 × 10^5^ MS5-hDLL4 cells were combined with 9 × 10^4^ to 4 × 10^5^ iNKT-HPC per ATO in 1.5-mL microcentrifuge tubes and centrifuged at 300 × *g* for 5 min at 4 °C in a swinging-bucket centrifuge. The supernatant was carefully removed, and the cell pellet was resuspended by brief vortexing. For each ATO, a 0.4-μM Millicell Transwell insert (EMD Millipore, Billerica, MA; Cat. PICM0RG50) was placed in a 6-well plate containing 1.5 mL ATO culture medium per well. To plate the ATOs, the inserts were removed and rested on the edge of the plate to drain the excess medium. The cell slurry was adjusted to 15 μL per ATO and was gently deposited onto the cell insert. The medium was changed completely every 3–4 d.

### Flow cytometry and antibodies

Flow cytometry was performed using 2% FBS in PBS for 20 min on ice. The antibodies were added to cells at a 1:50 final dilution. PI was added to all samples before analysis. The LSRII Fortessa and FACS Aria II (BD Biosciences, San Jose, CA, USA) were used for flow cytometry analysis and cell sorting, respectively, and the data were processed using FlowJo (Tree Star). Monoclonal antibodies (specific clones in parentheses) used for surface staining of the following molecules were obtained from BioLegend (San Diego, CA): CD3 (UCHT1), CD4 (RPA-T4), CD5 (UCHT2), CD8α (SK1), CD14 (HCD14), CD45 (HI30), CD56 (HCD56), CD69 (FN50), CD80 (2D10), CD83 (HB15e), CD86 (IT2.2), CD122 (TU27), CD150 (SLAM), CD161 (HP-3G10), CD163 (GHI/61), CD206 (15-2), CD314 (1D11), CD335 (9E-2), CD336 (P44-8), TCR Vα24Jα18 (6B11), HLA-DR (L243), CD1d (51.1).

### DC maturation assay and cytokine production of iNKT cells

Human monocyte-derived DCs were induced as described previously^3^. Briefly, CD14^+^ monocytes were isolated from PBMCs by positive magnetic cell sorting using CD14 microbeads (Miltenyi Biotec, Auburn, CA, USA) and cultured at 1.0 × 10^6^ cells/mL in the presence of rhGM-CSF and rhIL-4 (50 ng/mL each). On day 6, the non-adherent DCs were harvested and served as immature DCs. After 24 h of co-culturing of dendritic cells and iNKT cells, microscopic observations were performed. Three days later, DC maturation markers of dendritic cells were analyzed using flow cytometry. Culture supernatants were also collected after 3 d of co-culture to measure the production of human IL-12p70, IL-4, IL-2, IFN-γ, and TNF using BD Cytometric Bead Array Flex Set System (BD Bioscience).

### In vitro cytotoxicity assays

51Cr release assays were conducted to evaluate the cytolytic ability of effector cells. Target tumor cells were loaded with 1.85 MBq 51Cr for 1 h, and 5000 tumor cells were co-incubated with effector cells for 5 h at effector-to-target (E:T) ratios ranging from 20:1 to 2.5:1. The supernatants were harvested, and 51Cr release was quantified using a beta counter (PerkinElmer). Percent lysis was calculated as: %lysis = (experimental lysis−spontaneous lysis)/(maximal lysis−spontaneous lysis) × 100%, where maximal lysis was induced by incubation in a 2% Triton X-100 solution.

### Whole transcriptome sequencing using the ion AmpliSeq transcriptome human gene expression kit

Total RNA was extracted from 100,000 cells using the NucleoSpin RNA Kit (740902.250; Macherey-Nagel) according to the manufacturer’s instructions. RNA (1 ng) was reverse transcribed into complementary DNA using a SuperScript VILO cDNA Synthesis Kit (Life Technologies, 11754050). Next, the cDNA was amplified using the Ion AmpliSeq Transcriptome Human Gene Expression Core Panel. The quality of the amplified libraries was evaluated using an Agilent 2100 Bioanalyzer (Agilent Technologies) and quantified by qPCR using an Ion Library Quantitation Kit (Life Technologies, 4468802). Template libraries were loaded onto an Ion 540 Chip (Life Technologies, A27765) using the Ion Chef System and subsequently sequenced on an Ion 5SXL.

### Read alignment and differential gene expression analysis

AmpliSeq sequencing data were analyzed using the ampliSeq RNA plugin available on the Ion Torrent sequencing platform. This plugin uses the Torrent Mapping Alignment Program, which aligns raw Ion Torrent sequencing reads against a custom reference sequence containing the targeted transcripts included in the AmpliSeq gene expression kit. Differential gene expression analysis was performed using R after quality control, which included counts per million conversions, log transformation, and filtering of lowly expressed genes. Normalization was performed using the TMM normalization method.

### Preparation of human monocyte-derived M2-macrophages and CFSE assay

CD14^+^ monocytes were isolated from PBMCs by positive magnetic cell sorting using CD14 microbeads (Miltenyi Biotec, Auburn, CA, USA). To induce M2 macrophages, monocytes were first incubated with 10 ng/mL rhM-CSF for 6 d, followed by stimulation with 20 ng/mL rhIL-4 and 20 ng/mL rhIL-10 for 4 additional days.

T cells were isolated from PBMCs by negative magnetic cell sorting using Pan T cell microbeads (Miltenyi Biotec, Auburn, CA, USA). PBMCs were from the same donor in CD14^+^ and T cell preparations. To evaluate T cell division, T cells were labeled with CFSE (Cayman Chemical).

We then co-cultured 1 × 10^4 ^T cells stimulated by Dynabeads Human T-Activator CD3/CD28 (Thermo Fisher Scientific) with 4 × 10^4^ M2 macrophages and 4 × 10^4^ 3D-CD4^+^ iNKT cells and measured their CFSE dilution by flow cytometry after 3 days of culture without activation/expansion cytokines.

### Statistics and Reproducibility

Data analyses and representations were performed with Excel (Microsoft) and GraphPad Prism software. Statistical tests and the number of experiments and samples are specified in the figures or figure legends. Parametric data are presented as mean ± standard deviation (SD) or mean ± standard error of the mean (SEM) as indicated. P-values were calculated by ordinary one-way ANOVA with Tukey’s multiple comparison test for Figs. 1c, 4, 5c, by 2way ANOVA with Šídák’s multiple comparison test for Fig. 3b, by 2way ANOVA with Tukey’s multiple comparison test for Fig. 3c, and by unpaired t test for Fig. 5b. P values < 0.05 were considered to be statistically significant.

### Reporting summary

Further information on research design is available in the Nature Portfolio Reporting Summary linked to this article.

## Supplementary information

Supplementary Information

Description of Additional Supplementary Files

Supplementary Data 1

Reporting Summary

## Data Availability

Whole transcriptome sequencing data in this study are available at Gene Expression Omnibus (GEO) with accession number GSE311015. Source data underlying graphs can be obtained from Supplementary Data 1. The other datasets generated and analyzed during this study are available from the corresponding author upon reasonable request.
