# Supplementary material for: Artificial thymic organoid culture generates functional iPSC-derived CD4+ invariant natural killer T cells
Source: Commun Biol. 2026 Jan 9;9:185. doi: 10.1038/s42003-025-09462-1 (PMC12881471; doi:10.1038/s42003-025-09462-1)
Supplement: Supplementary file 1 — Supplementary Information [file 42003_2025_9462_MOESM1_ESM.pdf]

SUPPLEMETARY INFORMATION

Artificial Thymic Organoid culture generates functional iPSC-derived CD4+ invariant Natural Killer T cells

Sara Shiina, Tatsuki Ueda, Shoichi Iriguchi, Yasushi Uemura and Shin Kaneko

Supplementary Figures 1 – 2

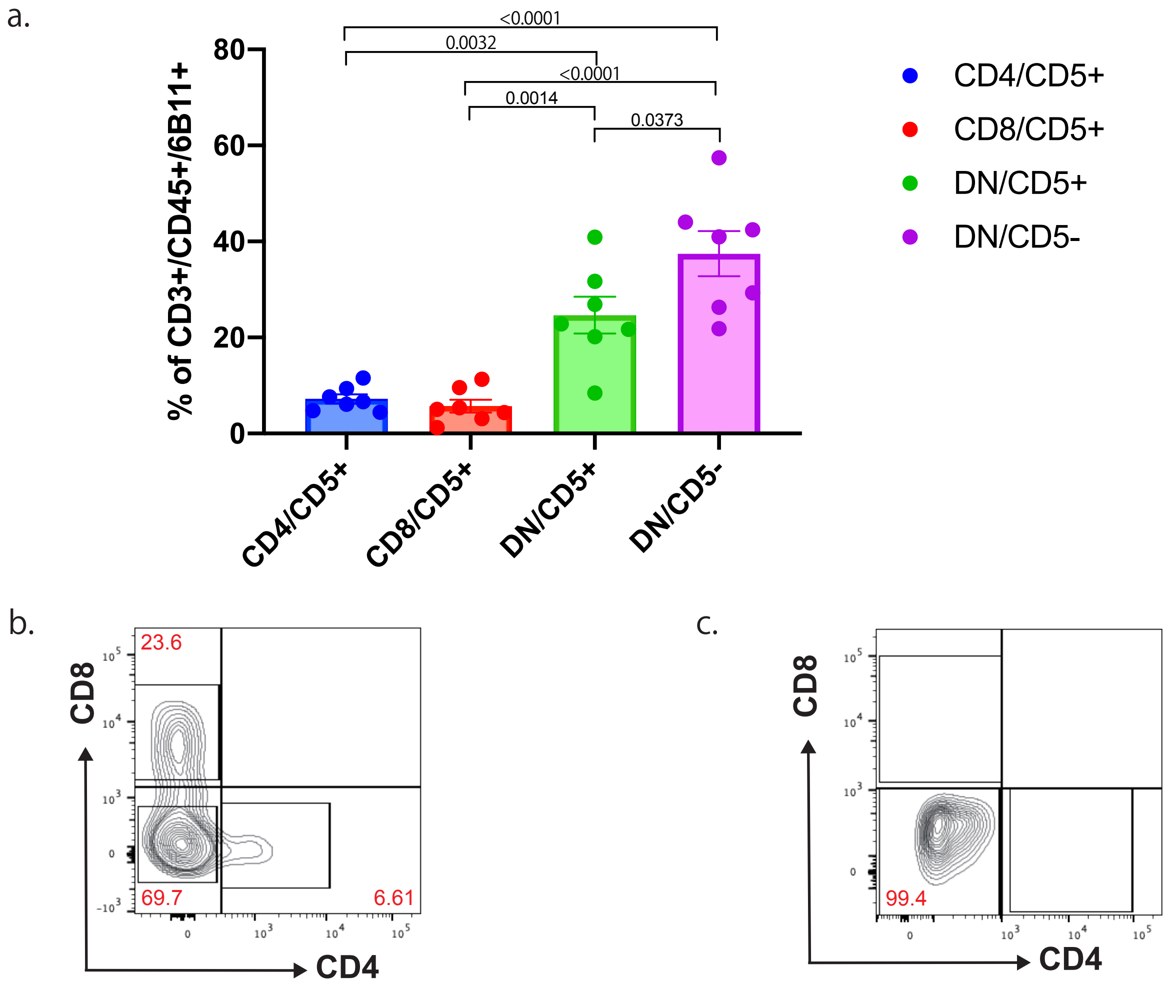

Supplementary Figure 1 : Induction of iNKT-cell differentiation from iNKT-iPS cells using ATO method

(a) Bar graphs is summarizing the percentage of each subset of 3D-re-iNKT cells for the seven trials (gated by CD45, CD3, and 6B11).

(b) Representative flow cytometry plots of re-differentiating T cells from CD4+iNKT-iPSC by ATO method (gated by CD45, CD3, and 6B11). (n=5)

(c) Representative flow cytometry plots of 3D-CD8 iNKT cells after expansion (gated by CD45, CD3, and 6B11). (n=5)

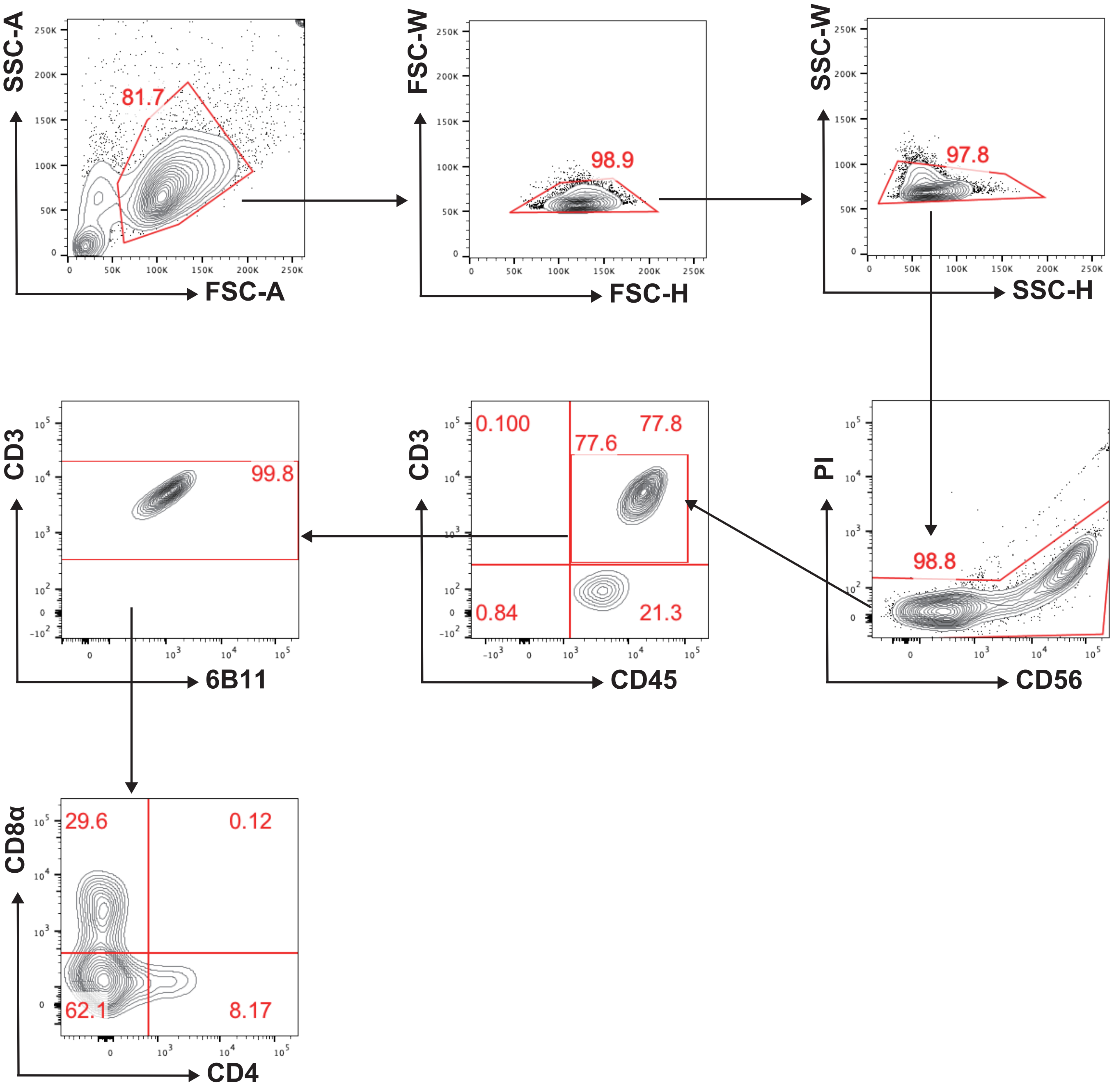

Supplementary Figure 2 : Gate Strategy

Gating strategy for iPS derived and primary iNKTcells. The data is a representative flow cytometry plot of 3D-re-iNKT cells.
