# Supplementary material for: Artificial thymic organoid culture generates functional iPSC-derived CD4+ invariant natural killer T cells
Source: Commun Biol. 2026 Jan 9;9:185. doi: 10.1038/s42003-025-09462-1 (PMC12881471; doi:10.1038/s42003-025-09462-1)
Supplement: Supplementary file 2 — Description of Additional Supplementary Files [file 42003_2025_9462_MOESM2_ESM.pdf]

# Description of Additional Supplementary Files

**File name:** Supplementary Data 1
**Description:** This is the dataset file including all source data underlying the graphs presented in the main/supplementary figures.
