# Supplementary material for: Artificial thymic organoid culture generates functional iPSC-derived CD4+ invariant natural killer T cells
Source: Commun Biol. 2026 Jan 9;9:185. doi: 10.1038/s42003-025-09462-1 (PMC12881471; doi:10.1038/s42003-025-09462-1)
Supplement: Supplementary file 4 — Reporting Summary [file 42003_2025_9462_MOESM4_ESM.pdf]

nature portfolio

Corresponding author(s): Shin Kaneko

Last updated by author(s): 11/21/2025

Reporting Summary

Nature Portfolio wishes to improve the reproducibility of the work that we publish. This form provides structure for consistency and transparency in reporting. For further information on Nature Portfolio policies, see our Editorial Policies and the Editorial Policy Checklist.

Statistics

For all statistical analyses, confirm that the following items are present in the figure legend, table legend, main text, or Methods section.

n/a	Confirmed	
☐	☒	The exact sample size (n) for each experimental group/condition, given as a discrete number and unit of measurement
☐	☒	A statement on whether measurements were taken from distinct samples or whether the same sample was measured repeatedly
☐	☒	The statistical test(s) used AND whether they are one- or two-sided<br>*Only common tests should be described solely by name; describe more complex techniques in the Methods section.*
☐	☒	A description of all covariates tested
☐	☒	A description of any assumptions or corrections, such as tests of normality and adjustment for multiple comparisons
☐	☒	A full description of the statistical parameters including central tendency (e.g. means) or other basic estimates (e.g. regression coefficient) AND variation (e.g. standard deviation) or associated estimates of uncertainty (e.g. confidence intervals)
☐	☒	For null hypothesis testing, the test statistic (e.g. F, t, r) with confidence intervals, effect sizes, degrees of freedom and P value noted<br>*Give P values as exact values whenever suitable.*
☒	☐	For Bayesian analysis, information on the choice of priors and Markov chain Monte Carlo settings
☒	☐	For hierarchical and complex designs, identification of the appropriate level for tests and full reporting of outcomes
☒	☐	Estimates of effect sizes (e.g. Cohen's d, Pearson's r), indicating how they were calculated

Our web collection on statistics for biologists contains articles on many of the points above.

Software and code

Policy information about availability of computer code

Data collection	FACS Diva 9.0.1 (Flow cytometry, CBA), VICTOR NIVO control software 5.0.0 (in vitro cytotoxicity assays), Torrent Suite 5.14 (RNAseq)
Data analysis	Flowjo 10.6.2, Prism 9, FCAP array 3.0.19.2091, Microsoft Excel for Mac 16.82

For manuscripts utilizing custom algorithms or software that are central to the research but not yet described in published literature, software must be made available to editors and reviewers. We strongly encourage code deposition in a community repository (e.g. GitHub). See the Nature Portfolio guidelines for submitting code & software for further information.

Data

Policy information about availability of data

All manuscripts must include a data availability statement. This statement should provide the following information, where applicable:

- Accession codes, unique identifiers, or web links for publicly available datasets
- A description of any restrictions on data availability
- For clinical datasets or third party data, please ensure that the statement adheres to our policy

The authors declare that all data that support the findings of this study are available in this article and are provided as a Source File Data file or from the corresponding author upon reasonable request.

Research involving human participants, their data, or biological material

Policy information about studies with human participants or human data. See also policy information about sex, gender (identity/presentation), and sexual orientation and race, ethnicity and racism.

Reporting on sex and gender	This information has not been collected.
Reporting on race, ethnicity, or other socially relevant groupings	This information has not been collected.
Population characteristics	This information has not been collected.
Recruitment	Recruited volunteers at university affiliated hospitals
Ethics oversight	Kyoto University Graduate School and Faculty of Medicine, Ethics Committee

Note that full information on the approval of the study protocol must also be provided in the manuscript.

Field-specific reporting

Please select the one below that is the best fit for your research. If you are not sure, read the appropriate sections before making your selection.

☒ Life sciences ☐ Behavioural & social sciences ☐ Ecological, evolutionary & environmental sciences

For a reference copy of the document with all sections, see nature.com/documents/nr-reporting-summary-flat.pdf

Life sciences study design

All studies must disclose on these points even when the disclosure is negative.

Sample size	No statistical methods were used to predetermine the experimental sample size. The sample size was determined based on prior literature in this field (e.g., Kennedy et al., Cell Reports, 2013).
Data exclusions	No data were excluded.
Replication	For all figures, two or three independent experiments were performed and all attempts at replicating observation were successful. The replication number is indicated in the legend of corresponding figures.
Randomization	All samples were number coded until the measurement was completed.
Blinding	Blinding was not performed. Fully blinded experiments were not possible due to personnel availability to accommodate such experiments.

Reporting for specific materials, systems and methods

We require information from authors about some types of materials, experimental systems and methods used in many studies. Here, indicate whether each material, system or method listed is relevant to your study. If you are not sure if a list item applies to your research, read the appropriate section before selecting a response.

Materials & experimental systems

n/a	Involved in the study	
☐	☒	Antibodies
☐	☒	Eukaryotic cell lines
☒	☐	Palaeontology and archaeology
☒	☐	Animals and other organisms
☒	☐	Clinical data
☒	☐	Dual use research of concern
☒	☐	Plants

Methods

n/a	Involved in the study	
☒	☐	ChIP-seq
☐	☒	Flow cytometry
☒	☐	MRI-based neuroimaging

Antibodies

Antibodies used	eFluor 450 anti-human CD3 UCHT1 invitrogen 48-0038-42<br>APC-Cy7 anti-human CD4 RPA-T4 BioLegend 300518<br>PE-Cy7 anti-human CD5 UCHT2 BioLegend 300622

PerCP-Cy5.5 anti-human CD8 SK1 BioLegend 344710
CD14 anti-human FITC HCD14 BioLegend 325604
FITC anti-human CD56 HCD56 BioLegend 318304
PE anti-human CD56 HCD56 BioLegend 318306
PE anti-human CD69 FN50 BioLegend 310906
FITC anti-human CD80 2D10 BioLegend 305206
Alexa Fluor 647 anti-human CD86 IT2.2 Biolegend 305416
Brilliant Violet 421 anti-human CD122 TU27 BioLegend 339010
FITC anti-human CD150 A12(7D4) Biolegend 306306
FITC anti-human CD158a/h HP-MA4 BioLegend 339503
PE anti-human CD158b DX27 BioLegend 312605
APC anti-human CD158e1 DX9 BioLegend 312716
APC anti-human CD161 HP-3G10 Biolegend 339912
PE-Cy7 anti-human CD161 HP-3G10 BioLegend 339918
APC-Cy7 anti-human CD163 GHI/61 BioLegend 333622
PE-Cy7 anti-human CD206 15-2 BioLegend 321123
PE-Cy7 anti-human CD314 1D11 BioLegend 320812
FITC anti-human CD335 9E2 BioLegend 331922
APC anti-human CD336 P44-8 BioLegend 325110
APC anti-human CD337 P30-15 BioLegend 325209
FITC anti-human TCR Vα24Jα18 6B11 eBioscience 11-5806-42
PerCP-Cy5.5 anti-human HLA-DR L243 BioLegend 307630
PE anti-human CD1d 51.1 BioLegend 350305

Validation

Antibodies were validated using positive and negative cells using human PBMCs.
Validation reports were also provided by the antibody manufacturers (BioLegend). Compensation controls were used for every experiment. The BD Fortessa and Aria Fusion were calibrated daily using CS&T beads (BD Biosciences).
https://www.thermofisher.com/antibody/product/CD3-Antibody-clone-UCHT1-Monoclonal/48-0038-42
https://www.biolegend.com/ja-jp/products/apc-cyanine7-anti-human-cd4-antibody-1933?GroupID=BLG5901
https://www.biolegend.com/ja-jp/products/pe-cyanine7-anti-human-cd5-antibody-4626?GroupID=BLG10091
https://www.biolegend.com/ja-jp/search-results/percp-anti-human-cd8-antibody-6321?GroupID=BLG10167
https://www.biolegend.com/ja-jp/products/fitc-anti-human-cd14-antibody-3951?GroupID=BLG11942
https://www.biolegend.com/ja-jp/products/fitc-anti-human-cd56-ncam-antibody-3795?GroupID=BLG15664
https://www.biolegend.com/ja-jp/products/pe-anti-human-cd56-ncam-antibody-3796
https://www.biolegend.com/ja-jp/products/pe-anti-human-cd69-antibody-1672?GroupID=BLG10251
https://www.biolegend.com/ja-jp/products/fitc-anti-human-cd80-antibody-552
https://www.biolegend.com/ja-jp/products/alexa-fluor-647-anti-human-cd86-antibody-3356?GroupID=BLG11941
https://www.biolegend.com/ja-jp/products/brilliant-violet-421-anti-human-cd122-il-2rbeta-antibody-8376?GroupID=BLG6338
https://www.biolegend.com/ja-jp/products/fitc-anti-human-cd150-slam-antibody-588?GroupID=BLG4484
https://www.biolegend.com/ja-jp/search-results/fitc-anti-human-cd158-kir2dl1-s1-s3-s5-antibody-5662?GroupID=BLG10197
https://www.biolegend.com/ja-jp/products/pe-cyanine7-anti-human-cd158b-j-kir2dl2-l3-s2-antibody-9648
https://www.biolegend.com/ja-jp/products/apc-anti-human-cd158e1-kir3dl1-nkb1-antibody-9688
https://www.biolegend.com/ja-jp/products/apc-anti-human-cd161-antibody-7420?GroupID=BLG10204
https://www.biolegend.com/ja-jp/products/pe-cyanine7-anti-human-cd161-antibody-7692?GroupID=BLG10204
https://www.biolegend.com/ja-jp/products/apc-cyanine7-anti-human-cd163-antibody-9866?GroupID=BLG10122
https://www.biolegend.com/ja-jp/products/pe-cyanine7-anti-human-cd206-mmr-antibody-7405
https://www.biolegend.com/ja-jp/explore-new-products/pe-cyanine7-anti-human-cd314-nkg2d-antibody-6499?GroupID=BLG8540
https://www.biolegend.com/ja-jp/products/fitc-anti-human-cd335-nkp46-antibody-8464?GroupID=BLG8494
https://www.biolegend.com/ja-jp/products/apc-anti-human-cd336-nkp44-antibody-3850?GroupID=BLG5086
https://www.biolegend.com/ja-jp/products/apc-anti-human-cd337-nkp30-antibody-3856
https://www.biolegend.com/ja-jp/products/fitc-anti-human-tcr-valpha24-jalpha18-inkt-cell-antibody-6029
https://www.biolegend.com/ja-jp/products/pe-anti-human-cd1d-antibody-6922?GroupID=BLG9098

Eukaryotic cell lines

Policy information about cell lines and Sex and Gender in Research

Cell line source(s)

K562 (JCRB Cell bank), MS5 (RIKEN Cell bank), C1R (KAC)

Authentication

None of the cell lines used have been authenticated.

Mycoplasma contamination

All cell lines were confirmed negative to mycoplasma contamination

Commonly misidentified lines
(See ICLAC register)

None of the cell lines used in this manuscript are listed in the ICLAC Database of Cross-contaminated or Misidentified Cell Lines.

Plants

Seed stocks

No plants was used in this study.

Novel plant genotypes

No plants was used in this study.

Authentication

No plants was used in this study.

Flow Cytometry

Plots

Confirm that:

☒ The axis labels state the marker and fluorochrome used (e.g. CD4-FITC).

☒ The axis scales are clearly visible. Include numbers along axes only for bottom left plot of group (a 'group' is an analysis of identical markers).

☒ All plots are contour plots with outliers or pseudocolor plots.

☒ A numerical value for number of cells or percentage (with statistics) is provided.

Methodology

Sample preparation

After washing, cells were stained with antibodies for 20 min on ice. PI was added to all samples before analysis. LSRII Fortessa and FACS AriaII (BD Biosciences, San Jose, CA) were used for flow cytometry analysis and cell sorting, respectively.

Instrument

FACS Aria II and LSRII Fortessa

Software

FACS Diva 9.0.1 and Flowjo 10.6.2

Cell population abundance

A portion of FACS or MACS-sorted cells was re-analyzed by flowcytometry (FACS Aria Fusion) and confirmed to > 97% purity for FACS-sorting and >90% for MACS-sorting, respectively.

Gating strategy

Gating was performed as follows: FSC/SSC -> Singlets -> Live cells (PI negative) -> gating of interest. The appropriate negative control was used for generating gates of interest. For instance, iNKT cell phenotypes were gated based on staining prepared by human PBMCs.

☐ Tick this box to confirm that a figure exemplifying the gating strategy is provided in the Supplementary Information.
